# Induced changes of pyrolysis temperature on the physicochemical traits of sewage sludge and on the potential ecological risks

**DOI:** 10.1038/s41598-020-79658-4

**Published:** 2021-01-13

**Authors:** Claudineia de Souza Souza, Marcela Rebouças Bomfim, Maria da Conceição de Almeida, Lucas de Souza Alves, Welder Neves de Santana, Itamar Carlos da Silva Amorim, Jorge Antonio Gonzaga Santos

**Affiliations:** Universidade Federal Do Recôncavo da Bahia, Rua Rui Barbosa, 710, Cruz das Almas, BA Brazil

**Keywords:** Chemistry, Environmental sciences, Environmental chemistry, Pollution remediation

## Abstract

Biochar from sewage sludge is a low-cost sorbent that may be used for several environmental functions. This study evaluates the induced effects of pyrolysis temperature on the physicochemical characteristics of sewage sludge (SS) biochar produced at 350 (SSB_350_), 450 (SSB_450_) and 600 (SSB_600_), based on the metal enrichment index, metal mobility index (MMI), and potential ecological risk index (PERI) of Cd, Cu, Pb, and Zn. Increased pyrolysis temperature reduced the biochar concentration of elements that are lost as volatile compounds (C, N, H, O, and S), while the concentration of stable aromatic carbon, ash, alkalinity, some macro (Ca, Mg, P_2_O_5_, and K_2_O) and micronutrients (Cu and Zn), and toxic elements such as Pb and Cd increased. Increasing the pyrolysis temperature is also important in the transformation of metals from toxic and available forms into more stable potentially available and non-available forms. Based on the individual potential ecological risk index, Cd in the SS and SSB_450_ were in the moderate and considerable contamination ranges, respectively. For all pyrolysis temperature biochar Cd was the highest metal contributor to the PERI. Despite this, the potential ecological risk index of the SS and SSBs was graded as low.

## Introduction

Biochar is a stabilized, recalcitrant organic carbon compound produced by pyrolysis^1^ when biomass is heated under low oxygen concentrations^2^ to temperatures usually between 300 and 1000 °C. The feedstock source, characteristics of the pyrolysis process largely determine the suitability of biochar for a given application^3^. Biochar physical (specific surface area, pore size and distribution); chemical (presence of functional groups, such as carboxyl, hydroxyl, phenolic, and aromatics^4^, and nutritional (phosphorus, nitrogen, sulphur, and micronutrients) characteristics^5^ are largely controlled by the raw material source and pyrolysis process. The diversity of physicochemical characteristics of biochar has allowed the tailoring of the material for use as an agricultural amendment^6^, for sequestration of carbon, and as a sorbent for potentially hazardous organic and inorganic compounds in aquatic environment, soil, and sediments^7^. It also has applications in mitigation of climate change^8^, energy production^3,9^ industry, and engineering^10^. One of the reasons for the wide acceptance of biochar use is the possibility of reuse of different biomass sources, such as garbage or refuse, sludge from waste treatment plants, and discarded material resulting from industrial, commercial, mining, agricultural, and community activities^11^.

The disposal of sewage sludge (SS) in landfills and by incineration has been gradually replaced by the use of the material for biochar production. Use of traditional disposal methods has reduced due to land limitations, secondary contaminant production, and the risk of polluting farmland and surface or subsurface water. Solid SS from wastewater treatment has received attention as a biomass source for biochar production^12,13^, and is used in agriculture because of its high nitrogen, phosphorus, and micronutrient content. One of the advantages of using SS from waste water treatment plants (WWTPs) as a sustainable source for biochar production is its worldwide availability, and the tendency for its production to increase over time due to an increase in global population and the number of households connected to SS systems in developing countries.

The use of SS for biochar production mitigates the problems caused by SS waste volume; presence of harmful pharmaceuticals; pathogenic vectors and organisms; and potentially toxic elements (PTEs), and reduces the amount of carbon released to the atmosphere^12^. The resultant biochar is a pathogen-free material with great potential for immobilizing inorganic contaminants^13^, and PTEs are transformed into less toxic forms^14^.

Pyrolysis has been developed as a sustainable treatment technique for sludge management because it has the potential to simultaneously target energy recovery, nutrient recycling, heavy metal immobilization, and environmental protection^15^. The heating rate, residence time, and temperature have a direct impact on biochar characteristics^16^. Biochar produced at high temperatures has a high carbon content, large surface area, and low reactivity functional groups, as indicated by the low H/C and O/C molar ratios, reflecting the loss of highly degradable compounds through dehydration and decarboxylation reactions^17^.

Although biochar production and use offer many opportunities for enhancing soil and environmental health quality, for use on a large scale, individual and environmental safety protocols must be adopted to regulate feedstock quality, pyrolysis processes, and certification of the final product to indicate its suitability for further use.

Garbage or refuse, sludge from waste treatment plants, and material discarded during industrial, commercial, mining, agricultural, and community activities may contain toxic levels of PTEs^18^, polycyclic aromatic hydrocarbons (PAH), polychlorinated dibenzodioxins (PCDDs), polychlorinated dibenzofurans (PCDFs) polychlorinated biphenyl (PCB), inorganic pesticides, dioxins, persistent organic pollutants (POPs)^19,20^, toxins^21^; glass, hard plastic, film plastic, metals, textiles^22^; and polyvinyl chloride (PVC)^23^. Protection against cytotoxicity caused by fine biochar particles commonly released during biochar production and field application^24^ is a further health risk that needs to be assessed.

The total PTE concentration in the biochar matrix is a useful pollution indicator of content but it provides no information on the metals environment impact, which depends on their chemical form^25^. The data produced by the chemical speciation or sequential extraction allows to determine the mobile, bioavailable PTEs forms in the sludge and biochar^26^ which may limit the disposal and utilization due to their environmental risk.

The potential ecological risk index (PERI) proposed by Ref.^27^ may be used as an important tool to evaluate the detrimental effect of PTEs present in the biochar biomass to the microbial populations on plants, animals, and humans^28^. The PERI index integrates a single-element potential ecological risk (Er) for all PTEs present in a sample^18^. Despite the need to evaluate other ecotoxicological factors that may restrict the use of biochar in the environment, this study was undertaken with the specific objectives of evaluating the effect of pyrolysis temperature on SS biochar: (1) physicochemical characteristics, (2) availability of PTE, defined here as Cd, Cu, Pb, and Zn, and (3) the potential ecological risk as a means of providing a scientific basis for the safe and eco-friendly use of the material.

## Materials and methods

### Biochar production

The SS used in this study was derived from the WWTP of the Bahian Water and Sanitation Company (EMBASA) in Cruz das Almas, Bahia State, Brazil. The feedstock was air-dried and then pyrolyzed at temperatures of 350, 450 and 600 °C, and the resultant biochar is hereafter referred to as SSB_350_, SSB_450_, and SSB_600_, respectively. The pyrolysis process of the sludge was carried out by a third-party company (Bahiacarbon Agroindustrial LTDA) at a rate of 10 °C min^−1^ until the target temperature was reached, with an average yield of 5–10%. The final temperature was maintained for 2 h; the sample was then cooled slowly to room temperature. The SSB was homogenized and screened to pass through a 2 mm stainless sieve.

### Ultimate and proximate analysis

The ultimate analyses (C, H, N, and S concentrations) of the sewage sludge (SS) and sewage sludge biochar (SSB) were determined using an automatic elemental analyzer (Vario EL III, Elementar, Hanau, Germany). The percentage of oxygen in the samples was calculated according to the formula 1:

1 $${\text{O}} \left( \% \right) = 100 - \left( {{\text{C}}\,\% + {\text{H}}\,\% + {\text{N}}\,\% + {\text{S}}\,\% + {\text{ash}}\,\% } \right)$$

The proximate analysis [moisture, ash, volatiles, and fixed carbon (FC)] of the sewage sludge (SS) and sewage sludge biochar (SSB) were determined using different procedures. The moisture content was determined by the weight loss of the sample as it was heated to 150 °C. The volatile content was determined as the sample was heated from 150 to 750 °C in a muffle (Linn-Elektro Therm model N 480 D). The ash content of each sample was measured by dry combustion in a muffle furnace at 750 °C for 6 h^29^. Fixed carbon was calculated according to the formula 2:

2 $${\text{FC}} \left( \% \right) = 100\% - \left( {{\text{moisture}}\,\% - {\text{volatile}}\;{\text{matter}}\,\% - {\text{ash}}\,\% } \right)$$

### Characteristics of the mineral fractions

The total metal concentration in the biochar was determined in 5 cm of macerated and homogenized samples deposited in 20 cm acrylic capsules. sealed with a 0.2 mm thick polypropylene film. The samples were analyzed by the method 6200^30^ using energy dispersive portable X-ray fluorescence (PXRF) spectrometry (Brucker, model Titan 600).

The metal concentration was also determined in a 3050B extract^31^ by atomic absorption spectroscopy (Varian, model FS 240F). Samples of 0.5 g of SS and SSB were digested in 5 mL of 2 M HNO_3_ solution together with 2 mL H_2_O_2_ (30%) and the volume was made up to 50 mL with Milli-Q water.

### Physicochemical characteristics

The feedstock and SSB samples were suspended in deionized water and 1 M KCl (1:10 m/v ratio), stirred for 30 min and allowed to stand for 5 min, then assessed for pH_(H2O)_ and pH_(KCl)_, respectively, using a pH meter (Hanna, model HI 3221). The electroconductivity (EC) was analyzed using a conductivity meter (Tecnal, model 4 MP).

Cation exchange capacity (CEC) was measured as described by Ref.^32^. In summary, 25 g of each biochar sample and 125 mL of 1 M NH_4_OAc were transferred into 200 mL vessels and shaken on a reciprocal shaker for 15 h. The vessel contents were poured through a filter paper-fitted Buchner funnel. Each flask containing biochar was rinsed four times with 25 mL NH_4_OAc to remove biochar stuck onto container sides and the leachate was discarded. The biochar on the filter paper was rinsed eight times by adding 25 mL of 95% CH_3_CH_2_OH to remove the excess NH_4 adsorbed_. The NH_4_^+^ adsorbed in the biochar was displaced with 1 M KCl. The leachate was transferred to a 250 mL volumetric flask and the volume was made up to 250 mL with 1 M KCl. The concentration of NH_4_^+^ in the KCl extract was determined by Spectro colorimetry analysis (PerkinElmer Model Lambda 25 UV/Vis) at ƛ = 400 nm. The concentration of NH_4_^+^ was determined in both the sample and the blank KCl extraction solution. The concentration of NH_4_^+^ was calculated using the Nessler method^33^ according to Eq. (3):

3 $$CEC = \frac{{NH4^{ + } \;{\text{Extractant}} - NH4^{ + } \;{\text{Blank}}}}{14}$$

where CEC is the cation exchange capacity (cmol_c_ kg-^−1^); NH_4_^+^Extractant is the concentration of NH_4_^+^ adsorbed by the biochar (mg L^−1^) and NH_4_^+^ Blank is the concentration of NH_4_^+^ in the blank extractant solution (mg L^−1^).

### Speciation of potential toxic metals (PTEs)

The chemical speciation of PTEs (Cu, Cd, Pb, and Zn) in the SS and SSBs were carried out according to Tessier et al.^34^ tests. The water-soluble fraction (F1) was extracted with Milli-Q water for 18 h; the exchangeable fraction (F2) was extracted with 1 M MgCl_2_ at pH 7 for 1 h; the carbonate fraction (F3) was extracted with 1 M CH_3_COONa at pH 5 for 5 h; the weakly crystalline Fe and Mn oxides fraction (F4) was extracted with 0.04 M NH_2_OH in 25% (V/V) CH_3_COOH (pH 2) for 6 h at 96 °C; the organic matter fraction F5 was extracted for 2 h at 85 °C with 3 mL of 0.02 M HNO_3_ and 5 mL of 30% H_2_O_2_ adjusted to pH 2 with HNO_3_, with occasional agitation; the residual fraction (F6) was extracted with 20 mL of 7 M HNO_3_ for 6 h at 80 °C. After each extraction, a washing step was performed to collect the remaining extractant solution. The solutions from the extraction and washing steps were combined for chemical analysis. After each extraction procedure, the mixture was centrifuged (Hettich 420R) at 3000 rpm for 30 min, and the supernatant was collected and filtered (< 0.45 mm). The metal content of each fraction was determined by atomic absorption spectroscopy (Varian FS 240F).

### Potential toxic metals and mobility enrichment index (MEI)

The metal enrichment index (MEI), ratio of the PTE concentration in the biochar sample (SSB), and the background metal concentration (for SS) were used to evaluate the influences of pyrolysis temperatures on SSB metal enrichment. The MEI was calculated using Eq. (4):

4 $$MEI = \frac{Ci \;biochar}{{Ci\;SS}}$$

where Ci biochar and Ci SS are the PTE concentrations where i (i.e., Pb, Cu, Cd, and Zn) is determined for the biochar produced at a given pyrolysis temperature, and in the SS, respectively. The metal mobility index (MMI) of the PTEs in the SS and SSBs measuring the metal availability and mobility in SS and biochar was calculated according to Kabala and Singh^35^, Eq. (5). The most labile fraction (F1, F2, and F3) of each metal was divided by the total metal concentration (F1 + F2 + F3 + F4 + F5 + F6).

5 $$MMI = \frac{F1 + F2 + F3}{{F1 + F2 + F3 + F4 + F5 + F6}} \times 100$$

### Potential ecological risk index (PERI)

The potential ecological risk index (PERI) proposed by Hakanson^27^ was used to evaluate the potential ecological risk of PTEs in biochar produced at different temperatures. The method takes into consideration the toxic level, total concentration, and ecological sensitivity to PTEs^36^. The PERI was calculated according to the steps described by Eqs. (6, 7 and 8):

6 $$Cf = \frac{Cm}{{Cn}}$$

7 $$ER = Tr*Cf$$

8 $$RI = \sum Er$$

where Cf is the contamination factor, a measure of the degree of pollution on PTE; Cm and Cn are the concentrations of each PTE in the mobile (F1 + F2 + F3) and stable fractions (F4 + F5 + F6) respectively; T_r_ is the biological toxic factor for individual metals: Zn (1), Cu (5), Pb (5), and Cd (30)^27^; E_R_ is the potential ecological risk index of a single element; PERI is the potential ecological risk index of the overall contamination. The values of Cf, E_R_, and PERI were used to assess the risk of metal in the SS and the different pyrolysis temperature SSB_s_.

### Experimental design

The study was carried out as a completely randomized design with four treatments (SS, SSB_350,_ SSB_450_, and SSB_600_), and four repetitions. One-way analysis of variance (ANOVA) was performed for all variables. For variables that present significant effects (*p* < 0.05), the means were separated using the least significant difference (LSD) test at *p* < 0.05. The computer-based software SPSS V25^37^ was used for descriptive statistical analyses. When necessary, the software was used to adjust the linear regression.

## Results and discussion

### Ultimate and proximate analysis

**Figure 1:**
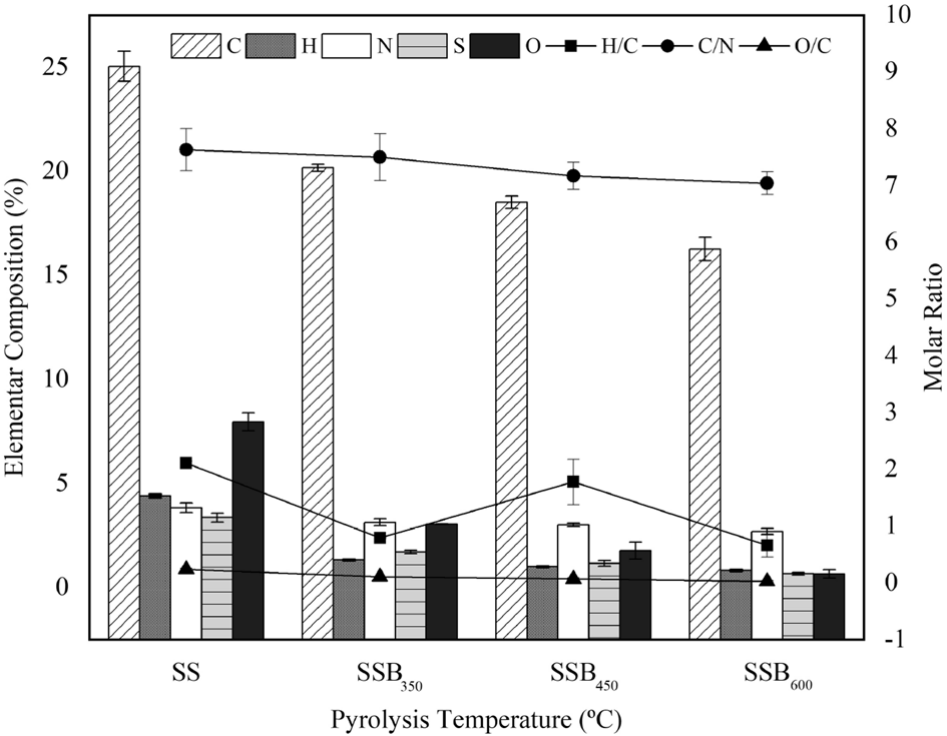
Ultimate analysis of sewage sludge (SS) and sewage sludge biochar (SSB) produced at the pyrolysis temperatures of 350 (SSB_350_), 450 (SSB_450_) and 600 (SSB_600_).

When compared to biochar produced from other feedstocks, sewage biochar is characterized by low C, N, and H values, especially C^38,39^. In this study, the C (C (%) = − 0.01 PYTemp + 25.10, R^2^ 0.98), N (%N = − 0.002 PYTemp + 3.832, R^2^ = 0.89) , H (H (%) = − 0.01 PYTemp + 4.14, R^2^ 0.92), O (O (%) = − 0.01 PYTemp + 7.76, R^2^ 0.98), and S (S (%) = − 0.01PYTemp + 3.342, R^2^ = 0.98) contents decreased with increasing pyrolysis temperature (PYTemp) (Fig. 1). The elementary content of C (25.04 ± 0.72%), N (3.84 ± 0.23%), H (4.41 ± 0.09%), S (3.37 ± 0.21%) and O (7.96 ± 0.43%) in the SS was higher than C (16.26 ± 0.56%), N (2.70 ± 0.15%), H (0.83 ± 0.03%), S (0.67 ± 0.06%), and O (0.66 ± 0.20%) of the SSB_600_.

The reduction of C and O in the biochar occurs mostly by the volatilization of the elements as CO, CO_2_, H_2_O, and hydrocarbon during pyrolysis^40^. Additional loss of O alone or associated with H occurs during pyrolysis due to the reduction of the hydroxyl (–OH) functional groups, dehydration, and condensation processes^41^. Decreases in the H content with increasing pyrolysis temperature have also been reported for other feedstocks^5,12^. Nitrogen is lost mainly by the volatilization of different nitrogen groups such as NH_4_–N or NO_3_–N at low temperatures^42^, and pyridine at temperatures > 600 °C^43^. The decrease in S with temperature has been reported in other studies^44,45^. The loss of S from the biochar is due to sulfur containing volatile organic compounds. Organic sulfur losses to the vapor phase during pyrolysis have been primarily identified as carbonyl sulfide^46^.

The reflex of the pyrolysis temperature on the reduction of C, H, N, O, and S is the change in key biochar treatments such as the H/C molar ratio, an index of the biochar aromaticity and stability; the O/C molar ratio, an index of polarity or the abundance of polar oxygen-containing surface functional groups^47^; and the C*/*N molar ratio, an index of inorganic N release from organic matter when biochar is incorporated into soils^48^.

**Figure 2:**
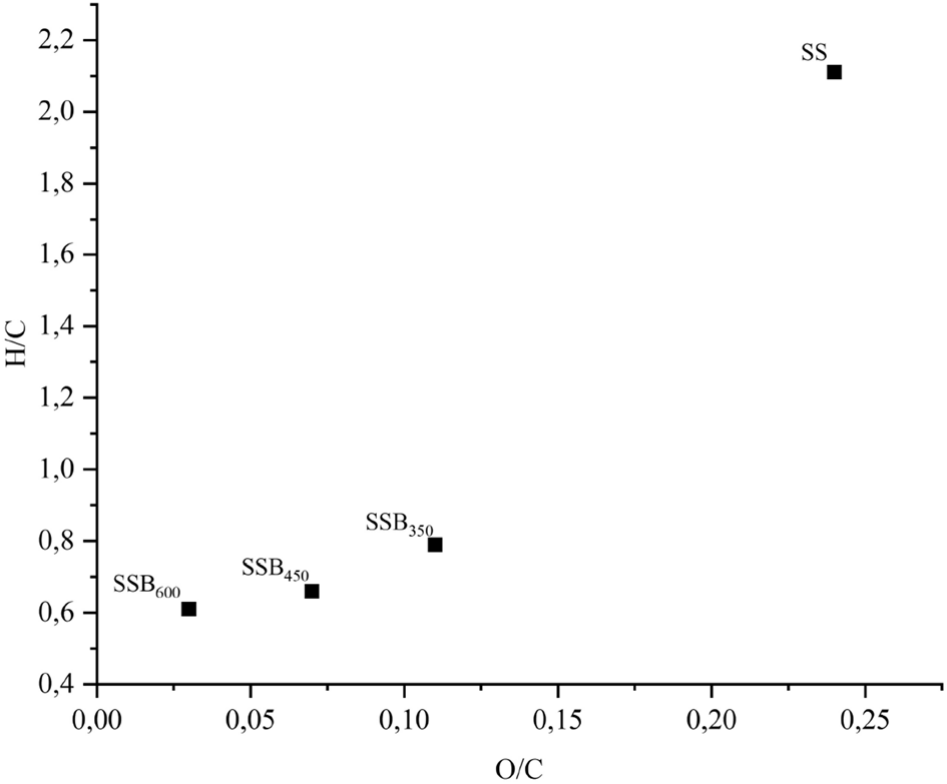
van Krevelen diagram for sewage sludge (SS) and sewage sludge biochar (SSB) produced at the pyrolysis temperatures of 350 (SSB_350_), 450 (SSB_450_) and 600 (SSB_600_).

The H/C and O/C molar ratios reduced with pyrolysis temperature and ranged from 2.11 ± 0.04 (SS) to 0.61 ± 0.02 (SSB_600_), and from 0.24 ± 0.02 (SS) to 0.03 ± 0.01 (SSB_600_). Plotting the H/C and O/C molar ratios in a van Krevelen diagram (Fig. 2) shows the reduction of the molar ratio with the increase of pyrolysis temperature. This result is in agreement with the results reported by Zhang et al.^49^, where evaluation of biochar from cow manure produced at 300, 400, 500, 600, and 700 °C also reported H/C and O/C molar reduction with increase of temperature and attributed the results to the formation of stable aromatic structures. According to Ahmad et al.^9^, at pyrolysis temperatures up to 480 °C, the decrease in H/C and O/C molar ratio with increasing temperature is due to the loss of carboxylic and phenolic functional groups that are responsible for the CEC; above 480 °C, the reduction occurs due to the processes of dehydration and deoxygenation, which reduce H- and O-containing functional groups.

The C/N molar ratio of the SS (7.63 ± 0.37) did not differ from those of SSB_350_ (7.50 ± 0.37) and SSB_450_ (7.17 ± 0.24), but it was reduced in the SSB_600_ (7.04 ± 0.20) as a result of the formation of compounds rich in C and poor in volatile N^50,51^. According to Jindo et al.^52^, the biochar from lignocellulosic material ranged from 40 to 256 ;thus, the low C/N ratio of the biochar in this study revealed the SSB potential as a source of N for plants.

Proximate analysis typically involves the determination of volatile matter, moisture, fixed carbon (FC), and ash^53^. Pyrolysis reduced the volatile content by up to 83% (from SS 36.6 ± 0.7 to SSB_600_ 6.3 ± 0.3) due to the transformation of compounds containing O–C=O into gas^53^, leading to an increase in the concentration of Si, Al, and Fe oxides with increasing temperature. Working with sewage sludge pyrolyzed at temperatures from 300 to 900 °C^54^ also yielded a reduction in volatile compounds with increasing temperature. The moisture content of the SS (7.3 ± 0.0%) reduced by approximately five times as much as the SSB_600_ (1.5 ± 0.2%; Table 1). The moisture reduction was attributed to water evaporation and loss of pyrolytic volatiles^55^ relative to SS.

**Table 1:** Proximate analysis of sewage sludge (SS) and sewage sludge biochars (SSB) produced at 350 (SSB_350_), 450 (SSB_450_) and 600 (SSB_600_).

Biochar characteristic	SS	SSB_350°_	SSB_450°_	SSB_600°_
Moisture (%)	7.3 ± 0.0	2.7 ± 0.2	2.2 ± 0.0	1.5 ± 0.2
Ash (%)	55.4 ± 1.9	72.5 ± 2.6	74.5 ± 0.6	78.9 ± 0.7
Volatiles (%)	36.6 ± 0.7	19.0 ± 0.1	11.4 ± 0.3	6.3 ± 0.3
C fixo (%)	2.0 ± 0.4	6.7 ± 0.7	12.0 ± 0.7	13.2 ± 1.0

**Figure 3:**
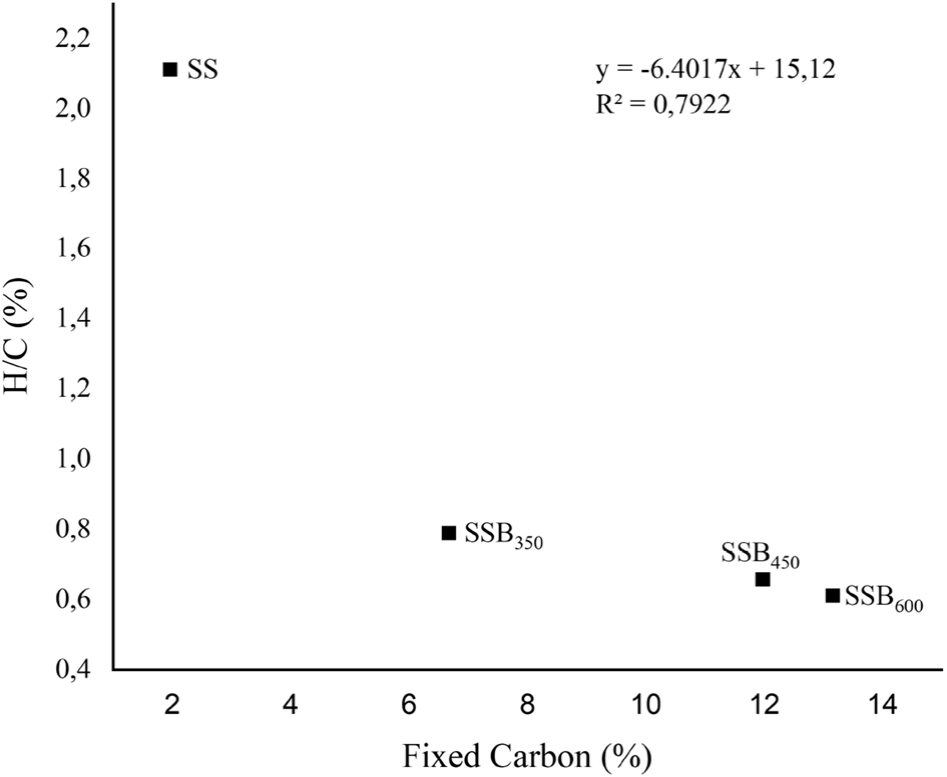
Correlation between ash and fixed carbon of sewage sludge (SS) and sewage sludge biochar (SSB) produced at the pyrolysis temperatures of 350 (SSB_350_), 450 (SSB_450_) and 600 (SSB_600_).

The content of FC, carbon remaining after loss of moisture and free volatile materials of the SSB_600_ (13.2 ± 1.0%) was about seven times higher than that obtained for SS (2.0 ± 0.4%). Working with biochar produced at different pyrolysis temperatures^5,47^ also found similar FC trends. The plot of H/C and FC content (Fig. 3) indicated that the SS material has more H relative to FC, while the biochar produced at high pyrolysis temperatures has less H relative to FC. Working with biochar derived from macaúba endocarp pyrolyzed at temperatures from 200 to 700 °C^56^, reported a similar relationship between H/C and FC. The reduction in the H/C ratio at higher temperatures also results from the breakup of oxygen-containing functional groups, such as carboxyl, carbonyl, and methoxyl, and the formation of aromatic compounds^57^.

Ash accounted for between 72.7% and 81.5% of the proximate analysis components (Table 1). The ash content in the biochar (SSB_350_ 72.5 ± 2.6%, SSB_450_ 74.5 ± 0.6% and SSB_600_ 78.9 ± 0.7%) was higher than in the SS (55.4 ± 1.9%; Table 1). These results are similar to the one reported by Regkouzas and Diamadopoulos^60^, that studying SSB produced at 300 (63.97%), 500 (77.44%) and 700 (81.15%) also found increased ash concentration with pyrolysis temperature and values closed to the ones found in this study. The pyrolysis process impacts not only the biochar ash concentration but also the quality of the material produced, which will be discussed in the following sections.

### Physicochemical characteristics

Pyrolysis at different temperatures promoted significant physicochemical changes in the feedstock (Table 2). When compared to feedstock pH (pH_H2O_ 4.5 and pH_KCl_ 4.2), the biochar acidity was reduced to 1.3 pH_H2O_ units (pH 4.8 SSB_350_, pH 5.7 SSB_450_, and pH 5.8 SSB_650_) and 1.2 pH_KCl_ units (pH 4.4 SSB_350_, pH 5.3 SSB_450_, and pH 5.4 SSB_650_) with increasing pyrolysis temperature. The increase in biochar pH with thermal treatment has been attributed to the loss of acidic functional groups (carboxyl, hydroxyl, or formyl) on the biochar surface^1^. The increase in biochar alkalinity is due to the separation of alkali elements (Ca, Mg, and K) from organic constituents during pyrolysis^45^, which contributes to the potential liming effect. When studying biomass from SS pyrolyzed at 300, 500, and 700 °C^60^, also reported an increase in pH with increasing pyrolysis temperature.

**Table 2:** Physical–chemical characteristics of sewage sludge (SS) and sewage sludge biochars (SSB) produced at 350 (SSB_350_), 450 (SSB_450_) and 600 (SSB_600_).

Biochar characteristic	SS	SSB_350°_	SSB_450°_	SSB_600°_
pH_H2O_ (1:10)	4.5 ± 0.1	4.8 ± 0.0	5.7 ± 0.1	5.8 ± 0.0
pH_KCl_ (1:10)	4.2 ± 0.1	4.4 ± 0.0	5.3 ± 0.0	5.4 ± 0.0
EC dSm^−1^	4.0 ± 0.0	2.2 ± 0.0	1.6 ± 0.0	1.5 ± 0.3
CEC cmol_c_ kg^−1^	6.0 ± 0.1	6.3 ± 0.1	6.4 ± 0.0	6.0 ± 0.1

Biochar EC, an estimator of the amount of total dissolved salts in the sample, is one of several biochar properties influenced by the feedstock source and pyrolysis conditions, such as temperature, residence time, and activation treatment^61,62^. The EC of the SS (4.0 ± 0.0 dS m^−1^) was higher than that observed for SSB_350_ (2.2 ± 0.0 dS m^−1^), SSB_450_ (1.6 ± 0.0 dS m^−1^), and SSB_600_ (1.5 ± 0.0 dS m^−1^; Table 2). During SS pyrolysis, the ash content increases, whereas the solubility of salts and metals decreases^63,64^. This occurs because the water-soluble concentrations of K^+^, Ca^2+^, Mg^2+^, and P increase in biochar produced up to 200 °C, but above that temperature it is likely that they will form crystals like whitlockite [(Ca, Mg)_3_(PO_4_)_2_]. At pyrolysis temperatures over 500 °C they will be incorporated into the silicon structure, forming less soluble salts^58,65^.

The cation exchange capacity (CEC) is one of the most important biochar characteristics because it indicates the potential of the material to attract positively charged ions per unit of mass^66^. In this study, the CEC of the SSB_350_ (6.3 ± 0.1 cmol_c_ kg^−1^) and SSB_450_ (6.4 ± 0.0 cmol_c_ kg^−1^) were higher than those obtained for both SS and SSB_600_ (6.0 ± 0.1 cmol_c_ kg^−1^; Table 2). Biochar produced at temperatures up to 480 °C tends to have higher CEC because some acidic oxygenated functional groups, such as phenolic acid and carboxyl groups, are retained^67^. In contrast, biochar produced at temperatures above 480 °C has lower CEC^68^.

### Mineral composition

The total concentrations of some mineral elements are shown in Table 3. The reduction of C, H, S, O, moisture, and volatile content with increasing temperature shows a positive correlation with the increase in concentrations of nonvolatile elements normalized for oxides, such as SiO_2_, Al_2_O_3_, Fe, CaO, and P_2_O_5_, which are the main mineral components of SS and SSBs. The SiO_2_ concentrations in SSB_450_ (43.49 ± 0.11%) and SSB_600_ (40.85 ± 0.11%) were higher than the values observed for SS (33.37 ± 0.09%) and SSB_350_ (33.30 ± 0.29%). In contrast, the increase in Al_2_O_3_ concentration in the biochar with increasing pyrolysis temperature can be described by linear regression (Al_2_O_3_% = 0.0097 (Temperature) + 7.3453, R^2^ = 0.86).

**Table 3:** Mean and standard deviation of the main inorganic components of the biochar as determined by fluorescence X-ray.

Inorganic components	Sewage sludge	SSB_350_	SSB_450_	SSB_600_	Biochar guidelines
CaO (%)	2.87 ± 0.00	2.61 ± 0.01	2.24 ± 0.01	2.26 ± 0.01	–
P_2_O_5_ (%)	2.06 ± 0.01	2.49 ± 0.01	2.54 ± 0.03	2.72 ± 0.05	–
MgO (%)	0.96 ± 0.13	1.11 ± 0.16	1.11 ± 0.00	1.04 ± 0.14	–
K_2_O (%)	0.37 ± 0.0	0.40 ± 0.00	0.47 ± 0.00	0.46 ± 0.01	–
Fe (%)	5.05 ± 0.02	5.78 ± 0.00	5.90 ± 0.00	6.18 ± 0.05	–
Zn (mg kg^−1^)	650 ± 30	960 ± 20	1090 ± 20	1120 ± 38	416–7400
Cu (mg kg^−1^)	290 ± 0	440 ± 10	510 ± 10	520 ± 10	143–6000
Mn (mg kg^−1^)	450 ± 30	510 ± 20	560 ± 30	570 ± 30	–
Pb (mg kg^−1^)	50 ± 10	60 ± 10	90 ± 10	80 ± 10	121–300
SiO_2_ (%)	33.37 ± 0.09	33.30 ± 0.26	43.49 ± 0.11	40.85 ± 0.11	–
Al_2_O_3_ (%)	7.64 ± 0.03	9.38 ± 0.12	12.82 ± 0.06	13.15 ± 0.13	–

The concentration of Al_2_O_3_ found in this study was lower than the 17.2% and 29.6% reported by Fan et al.^69^ in biochar produced from a cyclic activated sludge system (CSS) process and an applied membrane bioreactor (KSS). The higher Al content was attributed to the presence of inorganic solids from the WWTP. The presence of SiO_2_, Al_2_O_3_, and Fe in biochar is generally associated with the presence of soil material or chemicals used in the coagulation step of SS treatment^70^.

The main macronutrients present in the feedstock were CaO, P_2_O_5_, and MgO, and Fe, Zn, Mn, and Cu were the main micronutrients. The P_2_O_5_ concentration (from 2.06 ± 0.01% SS to 2.72 ± 0.05% SSB_650_), and K_2_O (from 0.37 ± 0.0% SS to 0.46 ± 0.01% SSB_600_) increased with pyrolysis temperature (Table 3). The effect of pyrolysis on the MgO content was negligible, and there is no clear explanation for CaO reduction (from 2.80 ± 0.00% SS to 2.24 ± 0.01% SSB_450_) with increasing temperature. The MEI of the macronutrients followed the sequence P_2_O_5_ > K_2_O > MgO > CaO (Table 4). The increase in metal enrichment with temperature is due to the decomposition of organic matter, which results in the release of the metals associated with organic compounds, and loss of volatile content^18^.

**Table 4:** Metal enrichment Index of the biochar produced at 350 (SSB_350_), 450 (SSB_450_) and 600 (SSB_600_) as compared to the feedstock.

Metal	CaO	MgO	K_2_O	Fe	Zn	Cu	P2O5	Mn	Pb	Al_2_O_3_
SSB_350_	0.91 ± 0.00	1.16 ± 0.08	1.07 ± 0.01	1.15 ± 0.00	1.48 ± 0.04	1.49 ± 0.04	1.21 ± 0.00	1.12 ± 0.13	1.13 ± 0.15	1.23 ± 0.02
SSB_450_	0.78 ± 0.00	1.17 ± 0.15	1.27 ± 0.01	1.17 ± 0.01	1.67 ± 0.04	1.76 ± 0.02	1.24 ± 0.02	1.22 ± 0.06	1.65 ± 0.32	1.68 ± 0.01
SSB_600_	0.79 ± 0.00	1.11 ± 0.23	1.22 ± 0.03	1.22 ± 0.01	1.73 ± 0.07	1.79 ± 0.02	1.32 ± 0.02	1.27 ± 0.16	1.46 ± 0.43	1.72 ± 0.01

Biochar produced from organic residues such as SS has the potential to present high concentrations of PTE, and their content increases with pyrolysis temperature as they form inorganic salts, hydroxides, oxides, and/or sulfides^18,71^. Similar findings were obtained in this study (Tables 4 and 5), in which the concentration and MEI values of Cu, Zn, Pb, Mn, and Fe increased in line with pyrolysis temperature due to the loss of volatile materials and moisture^3^, and the high boiling points of PTEs^72^.

**Table 5:** Effect of the pyrolysis temperature on contamination factor (Cf), potential ecological risk coefficient (Er) and potential ecological risk Index (PERI) of the sewage sludge (SS) and sewage sludge biochar produced at 350 (SSB_350_), 450 (SSB_450_) and 600 (SSB_600_).

Sample	Cf				Er				PERI
	Cd	Cu	Zn	Pb	Cd	Cu	Zn	Pb	
SS	1.39 ± 0,07	0.03 ± 0.01	1.01 ± 0.10	0.00 ± 0.00	41.65 ± 2.01	0.15 ± 0.01	1.01 ± 0.10	0.00 ± 0.00	42.81 ± 2.08
SSB_350_	1.22 ± 0.11	0.00 ± 0.00	0.21 ± 0.01	0.00 ± 0.00	36.57 ± 3.17	0.02 ± 0.00	0.21 ± 0.01	0.00 ± 0.00	36.80 ± 3.16
SSB_450_	3.64 ± 0.53	0.02 ± 0.00	0.12 ± 0.01	0.00 ± 0.00	109.28 ± 15.89	0.10 ± 0.01	0.12 ± 0.01	0.00 ± 0.00	109.50 ± 15.89
SSB_600_	0.00 ± 0.00	0.02 ± 0.00	0.10 ± 0.01	0.06 ± 0.01	0.00 ± 0.00	0.10 ± 0.00	0.10 ± 0.01	0.29 ± 0.04	0.51 ± 0.04

The concentration of PTEs in the biochar followed the sequence Fe (from 5.05 ± 0.02% SS to 6.18 ± 0.05% SSB_600_) > Zn (from 650 ± 30 mg kg^−1^ SS to 1120 ± 38 mg kg^−1^ SSB_600_) > Mn (450 ± 30 mg kg^−1^ SS and 570 ± 30 mg kg^−1^ SSB_600_) > Cu (from 290 ± 0 mg kg^−1^ SS to 520 ± 10 mg kg^−1^ SSB_600_; Table 4). The higher Fe concentration in the SS (5.05%) compared to the other PTEs is related to the addition of ferric chloride during sludge aerobic digestion^73^. The MEI separated the micronutrients into two groups; metals with higher atomic mass (Cu and Zn) were enriched in higher proportions than those of lower atomic mass (Mn and Fe). The PTE concentrations in the SS and biochar used in this study are in agreement with the pollutant control standard of the International Biochar Initiative Guidelines^74^ (Cu from 143 to 6000 mg kg^−1^ and Zn from 416 to 7400 mg kg^−1^).

The Pb concentration ranged from 50 mg kg^−1^ (SS) to 80 mg kg^−1^ (SSB_600_; Table 3), and the MEI ranged from 1.13 (SSB_350_) to 1.65 (SSB_450_). Based on frequency, toxicity, and potential exposure, Pb is the second most dangerous element behind arsenic (As)^75^. However, the Pb concentration was below the lower limit (121–300 mg kg^−1^) reported by IBI^74^. Moreover, despite the relevance of the data for content and enrichment of PTE, it must be considered that the estimation of the total metal content is insufficient to assess metal bioavailability, environmental risk, and toxicity, which are controlled by their chemical species rather than their absolute quantities in the samples^76,77^.

### Metal fractionation and metal mobility index

The geochemical forms of PTEs in the environment determine their bioavailability, ecotoxicity, diffusion in mobile forms, and consequently their fate in the environment^71^. Metal fractionation is the term used to identify and quantify the different operationally defined species forms or phases in which an element occurs^78^. The fractionation scheme used in this study for quantification of PTEs was based on operationally defined fractions for the following pools: water-soluble (F1), exchangeable (F2), carbonate, (F3), Fe–Mn oxides (F4), organic (F5), and residual (F6) fractions.

The process used in this study for determination of metal availability in the SS and SSBs was adapted from the classifications of Ref.^18^. The metals present in the F1, F2, and F3 fractions were classified as bioavailable because they are readily released to the environment. The metals in the F4 and F5 fractions were classified as potentially bioavailable because they are leachable only under very rigorous conditions. The metals in F6 were classified as non-bioavailable because they are unlikely to leach and degrade under natural conditions.

**Figure 4:**
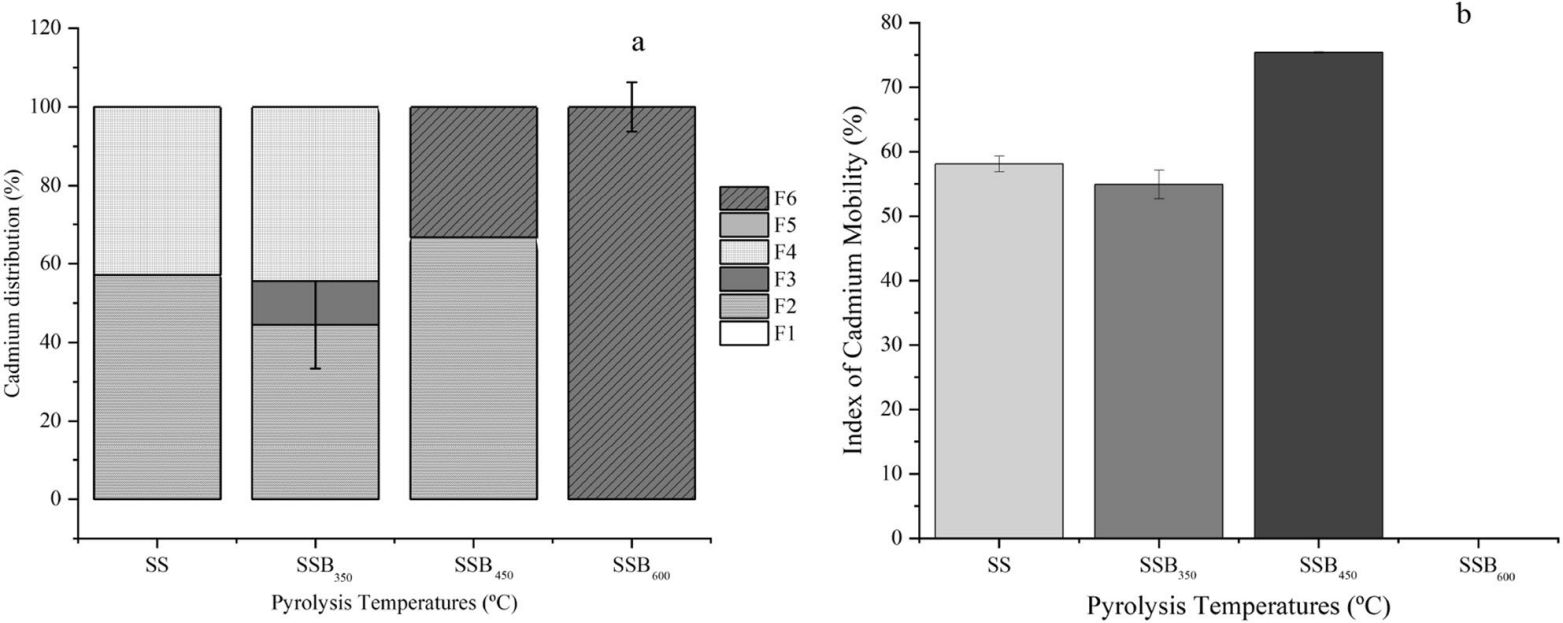
Cadmium fraction distribution (**a**) and mobility index (**b**) of sewage sludge biochar produced at 350 (SSB_350_), 450 (SSB_450_) and 600 (SSB_600_).

**Figure 5:**
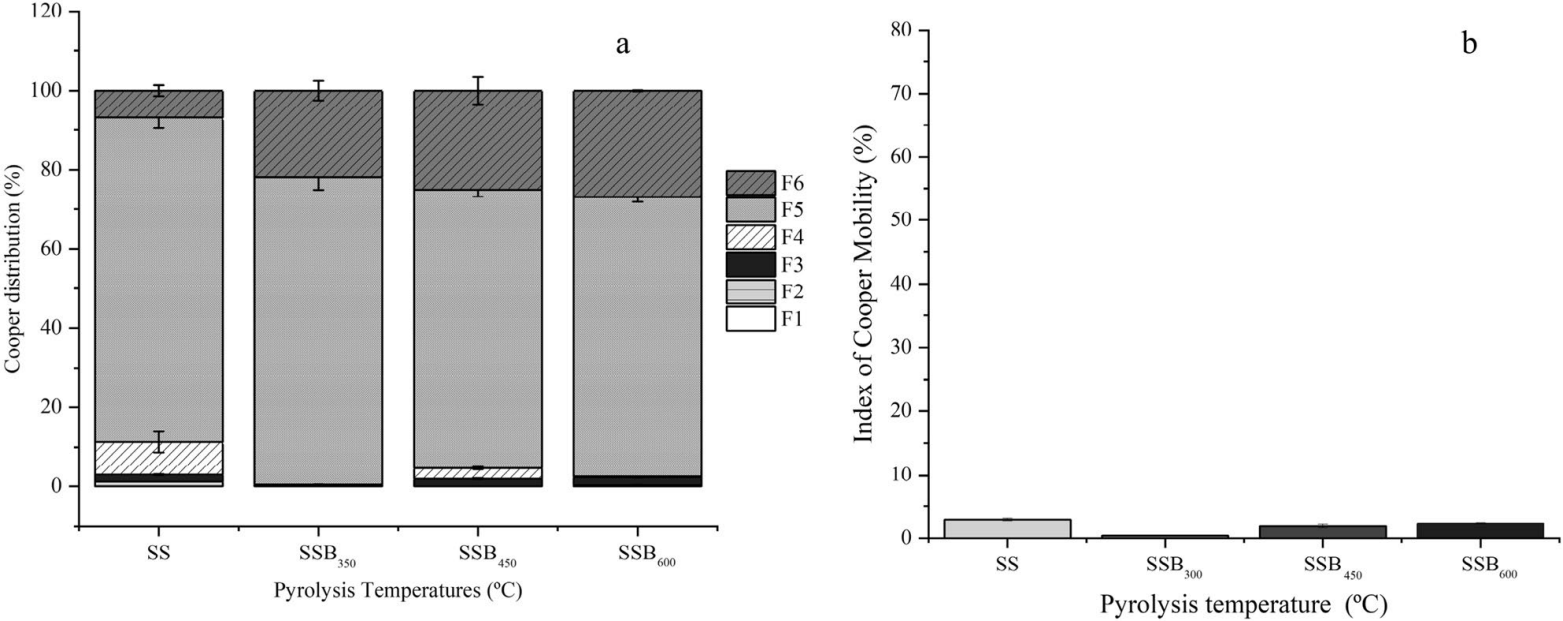
Cooper fraction distribution (**a**) and mobility index (**b**) of sewage sludge biochar produced at 350 (SSB_350_), 450 (SSB_450_) and 600 (SSB_600_).

**Figure 6:**
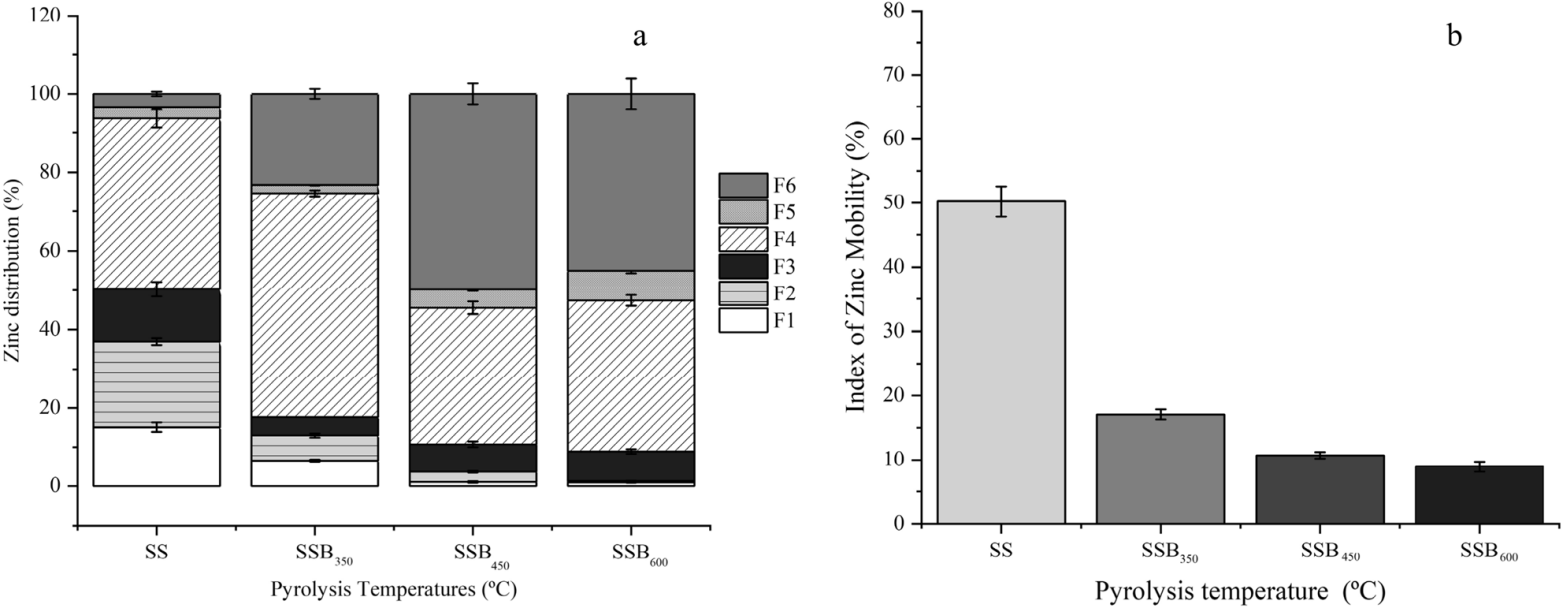
Zinc fraction distribution (**a**) and mobility index (**b**) of sewage sludge biochar produced at 350 (SSB_350_), 450 (SSB_450_) and 600 (SSB_600_).

**Figure 7:**
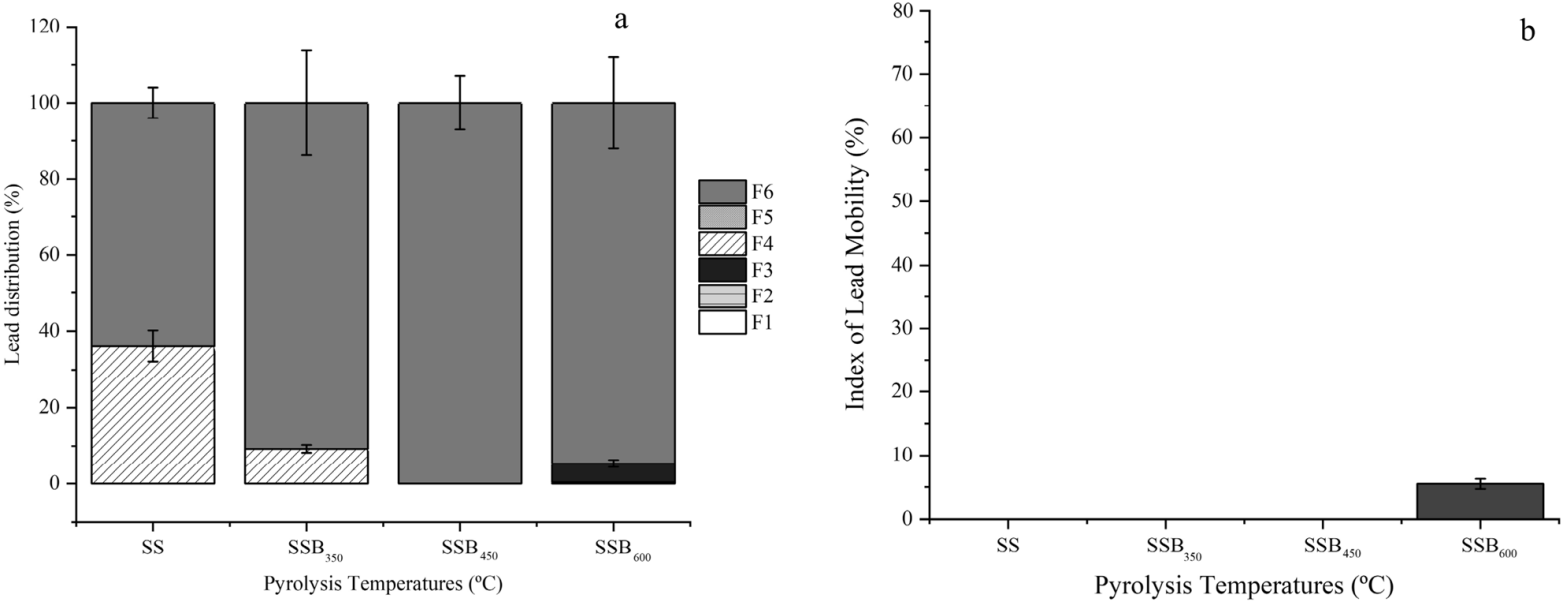
Lead fraction distribution (**a**) and mobility index (**b**) of sewage sludge biochar produced at 350 (SSB_350_), 450 (SSB_450_) and 600 (SSB_600_).

The metal concentrations resulting from USEPA 3050 are given by the sum of all fractions. The extractable fractions of the metals from the SS and SSBs, as well as their mobility index, are shown in Figs. 4, 5, 6 and 7. The total Cd concentration of the SSB_600_ (1.58 ± 0.02 mg kg^−1^) was ~ 2.5 fold higher than that of the SS (0.65 ± 0.02 mg kg^−1^; Fig. 4a). In contrast to Ref.^49^, where up to 73.3% of the Cd in cow manure biochar produced at 300, 400, 500, 600, and 700 °C was present in the directly toxic and bioavailable fraction, in this study, bioavailable and toxic forms represented 58.11 ± 1.19% (SS), to 54.86 ± 2.18% (SS_350_), and 75.41 ± 6.67% (SSB_450_). All Cd present in SSB_600_ was on the non-bioavailable form (Fig. 4a). Based on the MEI, the Cd present in SSB_600_ poses no risk to humans or microorganisms, whereas SS, SSB_350,_ and SSB_450_ have the potential to cause environmental toxicity (Fig. 4b).

The Cu concentration of SSB_600_ (470.14 ± 2.14 mg kg^−1^) was ~ 1.5-fold greater than that in the SS (329.53 ± 4.21 mg kg^−1^; Fig. 5a). Copper binds readily to organic constituents, forming a highly stable complex^79^. This was observed in this study; in the SS, 2.94 ± 0.22% of Cu was of the toxic bioavailable form, and 90.15 ± 1.37% was of the potentially bioavailable form (Fig. 5a). As the pyrolysis temperature increased, most of the Cu was distributed in potentially bioavailable (SSB_350_ 77.77 ± 2.12%, SSB_450_ 73.07 ± 2.47%, and SSB_600_ 70.69 ± 0.29%) and non-bioavailable (SSB_350_ 21.81 ± 2.19%, SSB_450_ 24.96 ± 2.64%, and SSB_600_ 27.01 ± 0.34%) forms. These results are similar to those reported by Lu et al.^80^ where study of SSBs produced at 300, 400, 500, 600, and 700 °C showed that most of the Cu was concentrated in the potentially available and non-available fractions. In this study, 96.3–100% of the potentially available Cu was in the organic fraction. The Cu enrichment in the biochar increased in line with the pyrolysis temperature (SSB_350_ 0.42%, SSB_450_ 1.92%, and SSB_600_ 2.31%); however, since most of the metal is of potentially bioavailable and non-bioavailable forms, this enrichment is not associated with its increase in ecotoxicity (Fig. 5b). Moreover, the Cu MEI in the SSBs was lower than that observed in the SS.

Zinc content in the SSB_600_ (665.82 mg kg^−1^) was 1.4 times higher than the SS (487.40 mg kg^−1^), and its distribution in the biochar fraction was greatly influenced by the pyrolysis temperature (Fig. 6a). The bioavailable Zn reduced with increasing temperature, which facilitated the transformation of the metal to the potentially bioavailable (SS 46.32 ± 2.51, SSB_350_ 59.25 ± 0.24, SSB_450_ 39.48 ± 0.26, and SSB_600_ 46.10 ± 1.76%) and residual (SS 3.43 ± 0.57, SSB_350_ 23.15 ± 0.89, SSB_450_ 49.84 ± 0.49, and SSB_600_ 45.02 ± 2.52%) forms. Between 83.5% and 96.3% of the potentially bioavailable Zn was in the reduced oxide fraction. The Zn MEI was 17.60 ± 0.83% for SSB_350_, 10.68 ± 0.54% for SSB_450_ and 8.88% ± 0.00 for SSB_600_ (Fig. 6b). According to Li et al.^81^, the low mobility of Cr, Ni, Cu, Zn, Cd, Pb, and Hg in sewage sludge biochar is due to their alkaline properties.

Lead content varied from 19.94 ± 1.52 mg kg^−1^ (SS) to 31.69 ± 3.83 mg kg^−1^ (SSB_600_). At least 91% of the Pb present in the SSBs was non-bioavailable (Fig. 7a). Similar to this study, evaluation by Wang et al.^59^ of biochar produced at temperatures of 300, 500, and 700 °C from hydrothermal pretreatment with pyrolysis (HTP) suggested that up to 99.97% of the Pb was in the residual fraction. Although Pb had the lowest MMI of all the metals studied, the index increased in line with temperature (SS 0.00 ± 0.00, SSB_350_ 0.00 ± 0.00, SSB_450_ 0.00 ± 0.00 and SSB_600_ 5.50 ± 0.78; Fig. 7b). The predominance of Pb in the residual fraction may be associated with the combination of the metal with primary minerals in the SS^59^.

### Risk analysis of potentially toxic elements

The risk to the environment and organisms posed by PTEs present in the SS and SSBs produced at different pyrolysis temperature was assessed by calculating the PERI (Table 5). Among the PTEs, Cd and Zn were the only metals that presented Cf values in the range of contamination. Cadmium Cf values increased in line with pyrolysis temperature, and the risk of contamination ranked from moderate for SS (1.39 ± 0.07) and SSB_350_ (1.22 ± 0.11), to considerable for SSB_450_ (3.64 ± 0.53), while Zn Cf values reduced in line with pyrolysis temperature, and only SS (1.01 ± 0.10) had a Cf value in the range for moderate contamination (Tables 5 and 6). The Pb and Cu Cfs were below the contamination values. According to Ref.^18^, the Cf of individual PTEs measures the degree of pollution by individual heavy metals, and its value is inversely proportional to its leaching potential.

**Table 6:** Grading of contamination factor (Cf), the potential ecological risk coefficient (Er) and potential ecological risk index (PERI).

Cf	Er	PERI	Ecological risk
< 1	≤ 40	PERi ≤ 150	Low contamination
1 < Cf ≤ 3	40 < Er ≤ 80	150 < Er ≤ 300	Moderate contamination
3 < Cf ≤ 6	80 < Er ≤ 160	300 < Er ≤ 600	Considerable contamination
6 < Cf ≤ 9	160 < Er ≤ 320	PERI > 600	High risk
Cf > 9	Er ≥ 320	–	Very high contamination

The index of potential ecological risk individuals, given by Er, is a function of the biological toxicity factor of individual PTEs^27^. Cadmium, present in the SS (41.65 ± 2.01) and SSB_450_ (109.28 ± 15.89), was the only metal with the individual potential ecological risk index value in the moderate (SS 41.65 ± 2.01) to considerable (SSB_450_ 109.28 ± 15.89) contamination range. The pyrolysis temperature had a significant effect on reduction of the Er values for the other metals studied (Table 5).

The potential ecological risk index (PERI) measured the degree of superposition of various harmful PTEs on organisms and the environment^81^. As reported in other studies^59,82^, the increase in pyrolysis temperature had a positive benefit on reducing the PERI value (SSB_350_ 36.80 ± 3.16%, and SSB_600_ 0.51 ± 0.04%) as compared with SS (42.81 ± 2.08%). The SSB_450_ (109.50% ± 15.89) presented a PERI value of 155.76%, higher than that for SS, because of the increase in Cd availability. Despite the SSB_450_ PERI value, the four PTEs have values that suggest a low potential ecological risk for utilization of biochar.

## Conclusions

The effects of pyrolysis temperature on sewage sludge biochar physicochemical properties were evaluated. The pyrolysis temperature affects the ultimate and proximate composition, the stability, aromaticity, and polarity of the biochar produced at different temperature. Moreover, the pyrolysis temperature also influenced the concentration of inorganic macro (Ca, Mg, P_2_O_5_, and K_2_O), micronutrients (Cu and Zn), and some toxic elements such as Pb and Cd. The pyrolysis temperature also has an important contribution in the transformation of metals from more toxic and available forms into more stable nontoxic and non-available forms. Based on the individual potential ecological risk index, Cd in the SS and SSB_450_ was in the moderate and considerable contamination ranges, respectively, and was the metal with the highest contribution to the PERI. Despite this contribution, the potential ecological risk index of the SS and SSBs was graded as low-risk.

## References

[1] Weber K, Quicker P (2018). Properties of biochar. Fuel.

[2] Jeffery S, Verheijen FGA, Van Der Velde M, Bastos AC (2011). A quantitative review of the effects of biochar application to soils on crop productivity using meta-analysis. Agric. Ecosyst. Environ..

[3] Chan KY, Xu Z, Lehmann J, Joseph S (2009). Biochar para o meio ambiente ciência e tecnologia de gestão. Biochar: Propriedades dos nutrientes e seus Aprimoramento.

[4] Frišták V, Soja G (2015). Effect of wood-based biochar and sewage sludge amendments for soil phosphorus availability. Nov. Biotechnol. Chim..

[5] Hossain MK, Strezov V, Chan KY, Ziolkowski A, Nelson PF (2011). Influence of pyrolysis temperature on production and nutrient properties of wastewater sludge biochar. J. Environ. Manag..

[6] Liu, C., Yang, J., Wang, G. & Ye, B. Impact of application of biochar-based fertilizer on the content of phosphorus and potassium in soil. In *IOP Conference Series: Earth and Environmental Science*. vol. 252 (2019).

[7] Jin J (2016). Cumulative effects of bamboo sawdust addition on pyrolysis of sewage sludge: biochar properties and environmental risk from metals. Bioresour. Technol..

[8] Lehmann J, Gaunt J, Rondon M (2006). Bio-char sequestration in terrestrial ecosystems—a review. Mitig. Adapt. Strateg. Glob. Change.

[9] Ahmad M (2014). Biochar as a sorbent for contaminant management in soil and water: a review. Chemosphere.

[10] Xie T, Reddy KR, Wang C, Yargicoglu E, Spokas K (2015). Characteristics and applications of biochar for environmental remediation: a review. Crit. Rev. Environ. Sci. Technol..

[11] Ndirangu SM, Liu Y, Xu K, Song S (2019). Risk evaluation of pyrolyzed biochar from multiple wastes. J. Chem..

[12] Agrafioti E, Bouras G, Kalderis D, Diamadopoulos E (2013). Biochar production by sewage sludge pyrolysis. J. Anal. Appl. Pyrolysis.

[13] Zielińska A, Oleszczuk P (2015). The conversion of sewage sludge into biochar reduces polycyclic aromatic hydrocarbon content and ecotoxicity but increases trace metal content. Biomass Bioenergy.

[14] Paz-Ferreiro J, Nieto A, Méndez A, Askeland MPJ, Gascó G (2018). Biochar from Biosolids Pyrolysis : A Review. Int. J. Environ. Res. Public Health.

[15] Chen T (2015). Adsorption of cadmium by biochar derived from municipal sewage sludge: impact factors and adsorption mechanism. Chemosphere.

[16] Qiang L, Wen-Zhi L, Xi-Feng Z (2009). Overview of fuel properties of biomass fast pyrolysis oils. Energy Convers. Manag..

[17] Spokas K (2010). Review of the stability of biochar in soils: predictability of O:C molar ratios. Carbon Manag..

[18] Devi P, Saroha AK (2014). Risk analysis of pyrolyzed biochar made from paper mill effluent treatment plant sludge for bioavailability and eco-toxicity of heavy metals. Bioresour. Technol..

[19] Camps Arbestain M, Saggar S, Leifeld J (2014). Environmental benefits and risks of biochar application to soil. Agric. Ecosyst. Environ..

[20] Schimmelpfennig S, Glaser B (2012). One step forward toward characterization: some important material properties to distinguish biochars. J. Environ. Qual..

[21] Kim HS (2015). Effect of biochar on heavy metal immobilization and uptake by lettuce (*Lactuca sativa* L.) in agricultural soil. Environ. Earth Sci..

[22] Brinton Junior WF (2005). Characterization of man-made foreign matter and its presence in multiple size fractions from mixed waste composting. Compos. Sci. Util..

[23] Cowie AL (2012). Is sustainability certification for biochar the answer to environmental risks?. Pesqui. Agropecu. Bras..

[24] Sigmund G (2017). Cytotoxicity of biochar: a workplace safety concern?. Environ. Sci. Technol. Lett..

[25] Su DC, Wong JWC (2003). Chemical speciation and phytoavailability of Zn, Cu, Ni and Cd in soil amended with fly ash-stabilized sewage sludge. Environ. Int..

[26] Flyhammar P (1998). Use of sequential extraction on anaerobically degraded municipal solid waste. Sci. Total Environ..

[27] Hakanson L (1980). An ecological risk index for aquatic pollution control—a sedimentological approach. Water Res..

[28] Schwarzenbach RP (2006). The challenge of micropollutants in aquatic systems. Science (80-).

[29] Enders A, Lehmann J, Singh B, Camps-Arbestain M, Lehmann J (2017). Proximate analyses for characterising biochars. Biochar: A Guide to Analytical Methods.

[30] USEPA (2007). Fiel portable X-Ray fluorescence spectrometry for the determination of elemental concentrations in soil and sediment. United States Environ. Prot. Agency.

[31] USEPA (1996). Method 3050B. Acid digestion of seidments, sludges, and soils. United States Environ. Prot. Agency.

[32] Aston S (2013). The impacts of pyrolysis temperature and feedstock type on biochar properties and the effects of biochar application on the properties of a sandy loam. Geophys. Res. Abstr..

[33] Tan LL, Ahmad M, Lee YH (2012). A novel optical ammonia sensor based on reflectance measurements for highly polluted and coloured water. Sens. Actuators B Chem..

[34] Tessier A, Campbell PGC, Bisson M (1979). Sequential extraction procedure for the speciation of particulate trace metals. Anal. Chem..

[35] Kabala C, Singh BR (2001). Fractionation and mobility of copper, lead, and zinc in soil profiles in the vicinity of a copper smelter. J. Environ. Qual..

[36] Douay F (2013). Assessment of potential health risk for inhabitants living near a former lead smelter. Part 1: metal concentrations in soils, agricultural crops, and homegrown vegetables. Environ. Monit. Assess..

[37] SPSS. *SPSS Statistics V25* (2017).

[38] Tripathi M, Sahu JN, Ganesan P (2016). Effect of process parameters on production of biochar from biomass waste through pyrolysis: A review. Renew. Sustain. Energy Rev..

[39] Qambrani NA, Rahman MM, Won S, Shim S, Ra C (2017). Biochar properties and eco-friendly applications for climate change mitigation, waste management, and wastewater treatment: a review. Renew. Sustain. Energy Rev..

[40] Lu T (2012). On the pyrolysis of sewage sludge: the influence of pyrolysis temperature on biochar, liquid and gas fractions. Adv. Mater. Res..

[41] Antal MJ, Gronli M (2003). The art, science, and technology of charcoal production. Ind. Eng. Chem. Res..

[42] Quicker P, Weber K (2016). Biokohle: Herstellung, Eigenschaften und Verwendung von Biomassekarbonisaten.

[43] Bagreev A, Bandosz TJ, Locke DC (2001). Pore structure and surface chemistry of adsorbents obtained by pyrolysis of sewage sludge-derived fertilizer. Carbon N. Y..

[44] Cantrell KB, Hunt PG, Uchimiya M, Novak JM, Ro KS (2012). Impact of pyrolysis temperature and manure source on physicochemical characteristics of biochar. Bioresour. Technol..

[45] Al-Wabel MI, Al-Omran A, El-Naggar AH, Nadeem M, Usman ARA (2013). Pyrolysis temperature induced changes in characteristics and chemical composition of biochar produced from conocarpus wastes. Bioresour. Technol..

[46] Ro KS, Cantrell KB, Hunt PG (2010). High-temperature pyrolysis of blended animal manures for producing renewable energy and value-added biochar. Ind. Eng. Chem. Res..

[47] Uchimiya M, Chang SC, Klasson KT (2011). Screening biochars for heavy metal retention in soil: role of oxygen functional groups. J. Hazard. Mater..

[48] Qayyum MF, Steffens D, Reisenauer HP, Schubert S (2014). Biochars influence differential distribution and chemical composition of soil organic matter. Plant Soil Environ..

[49] Zhang P, Zhang X, Li Y, Han L (2020). Influence of pyrolysis temperature on chemical speciation, leaching ability, and environmental risk of heavy metals in biochar derived from cow manure. Bioresour. Technol..

[50] Chen T (2014). Influence of pyrolysis temperature on characteristics and heavy metal adsorptive performance of biochar derived from municipal sewage sludge. Bioresour. Technol..

[51] Yuan JH, Xu RK, Zhang H (2011). The forms of alkalis in the biochar produced from crop residues at different temperatures. Bioresour. Technol..

[52] Jindo K, Mizumoto H, Sawada Y, Sonoki T (2014). Physical and chemical characterization of biochars derived from. Biogeosciences.

[53] Hu Y (2019). Thermal transformation of carbon and oxygen-containing organic compounds in sewage sludge during pyrolysis treatment. Energies.

[54] Zhang J (2015). Multiscale visualization of the structural and characteristic changes of sewage sludge biochar oriented towards potential agronomic and environmental implication. Sci. Rep..

[55] Neves D, Thunman H, Matos A, Tarelho L, Gómez-barea A (2011). Characterization and prediction of biomass pyrolysis products. Prog. Energy Combust. Sci..

[56] Guilhen SN, Mašek O, Ortiz N, Izidoro JC, Fungaro DA (2019). Pyrolytic temperature evaluation of macauba biochar for uranium adsorption from aqueous solutions. Biomass Bioenergy.

[57] Ma Z (2017). Evolution of the chemical composition, functional group, pore structure and crystallographic structure of bio-char from palm kernel shell pyrolysis under different temperatures. J. Anal. Appl. Pyrolysis.

[58] Cao X, Ma L, Liang Y, Gao B, Harris W (2011). Simultaneous immobilization of lead and atrazine in contaminated soils using dairy-manure biochar. Environ. Sci. Technol..

[59] Wang X, Chi Q, Liu X, Wang Y (2019). Influence of pyrolysis temperature on characteristics and environmental risk of heavy metals in pyrolyzed biochar made from hydrothermally treated sewage sludge. Chemosphere.

[60] Regkouzas P, Diamadopoulos E (2019). Adsorption of selected organic micro-pollutants on sewage sludge biochar. Chemosphere.

[61] Bird MI, Wurster CM, de Paula Silva PH, Bass AM, de Nys R (2011). Algal biochar–production and properties. Bioresour. Technol..

[62] Lu H (2013). Characterization of sewage sludge-derived biochars from different feedstocks and pyrolysis temperatures. J. Anal. Appl. Pyrolysis.

[63] de Figueredo NA, da Costa LM, Melo LCA, Siebeneichlerd EA, Tronto J (2017). Characterization of biochars from different sources and evaluation of release of nutrients and contaminants. Rev. Cienc. Agron..

[64] Tian Y, Cui L, Lin Q, Li G, Zhao X (2019). The sewage sludge biochar at low pyrolysis temperature had better improvement in urban soil and turf grass. Agronomy.

[65] Shinogi, Y. Nutriente leaching from carbon. In *ASAE/CSAE Annual International Meeting Meeting.* (2004).

[66] Bibar MPS (2014). Potencial Agrícola de Biocarvões Provenientes de Biomassas Alternativas.

[67] Mitchell PJ, Dalley TSL, Helleur RJ (2013). Preliminary laboratory production and characterization of biochars from lignocellulosic municipal waste. J. Anal. Appl. Pyrolysis.

[68] Novak JM (2009). Impact of biochar amendment on fertility of a southeastern coastal plain soil. Soil Sci..

[69] Fan J (2020). Using sewage sludge with high ash content for biochar production and Cu (II) sorption. Sci. Total Environ..

[70] Oh TK, Choi B, Shinogi Y, Chikushi J (2012). Effect of pH conditions on actual and apparent fluoride adsorption by biochar in aqueous phase. Water. Air. Soil Pollut..

[71] Callegari A, Hlavinek P, Capodaglio AG (2018). Production of energy (biodiesel) and recovery of materials (biochar) from pyrolysis of urban waste sludge. Rev. Ambient. Água.

[72] Wesenbeeck SV, Prins W, Ronsse F, Antal MJ (2014). sewage sludge carbonization for biochar applications. Fate Heavy Met..

[73] Barry D, Barbiero C, Briens C, Berruti F (2019). Pyrolysis as an economical and ecological treatment option for municipal sewage sludge. Biomass Bioenergy.

[74] International Biochar Initiative. Standardized product definition and product testing guidelines for biochar that is used in soil. *Int. Biochar Initiat.* 23 (2015).

[75] ATSDR. Substance Priority List | ATSDR. *Agency for Toxic Substances and Disease Registry* (2019). Available at: https://www.atsdr.cdc.gov/spl/index.html#2019spl. Accessed 7 April 2020.

[76] Yuan X (2011). Bioresource technology total concentrations and chemical speciation of heavy metals in liquefaction residues of sewage sludge. Bioresour. Technol..

[77] Huang H, Yuan X (2016). Bioresource technology the migration and transformation behaviors of heavy metals during the hydrothermal treatment of sewage sludge. Bioresour. Technol..

[78] Bogusz A, Oleszczuk P (2018). Sequential extraction of nickel and zinc in sewage sludge-or biochar/sewage sludge-amended soil. Sci. Total Environ..

[79] Shi W (2013). Bioresource technology immobilization of heavy metals in sewage sludge by using subcritical water technology. Bioresour. Technol..

[80] Lu Y (2015). Impacts of soil and water pollution on food safety and health risks in China. Environ. Int..

[81] Li F (2013). Spatial risk assessment and sources identi fi cation of heavy metals in surface sediments from the Dongting Lake, Middle China. J. Geochem. Explor..

[82] Leng L (2014). Bioresource technology the migration and transformation behavior of heavy metals during the liquefaction process of sewage sludge. Bioresour. Technol..

